# In Vitro Anti-Leishmanial Activity of Essential Oils Extracted from Vietnamese Plants

**DOI:** 10.3390/molecules22071071

**Published:** 2017-06-27

**Authors:** Thanh Binh Le, Claire Beaufay, Duc Trong Nghiem, Marie-Paule Mingeot-Leclercq, Joëlle Quetin-Leclercq

**Affiliations:** 1 GNOS Research Group, Louvain Drug Research Institue, Université Catholique de Louvain, Bruxelles 1200, Belgium; claire.beaufay@uclouvain.be; 2 Department of Pharmacognosy, Hanoi University of Pharmacy, 13-15 Le Thanh Tong, Hoan Kiem, Hanoi 100000, Vietnam; 3 Department of Botany, Hanoi University of Pharmacy, 13-15 Le Thanh Tong, Hoan Kiem, Hanoi 100000, Vietnam; ductrongeb@hup.edu.vn; 4 TFAR Research Group, Louvain Drug Research Institue, Université Catholique de Louvain, Bruxelles 1200, Belgium; marie-paule.mingeot@uclouvain.be

**Keywords:** *Leishmania mexicana mexicana*, essential oils, *Ocimum gratissimum*, *Cinnamomum cassia*, *Zingiber zerumbet*, *Elsholtzia ciliata*, *Amomum aromaticum*, eugenol

## Abstract

*Leishmania mexicana* is one of the pathogens causing cutaneous leishmaniasis which is associated with patient morbidity. In our researches for new safe and effective treatments, thirty-seven essential oils (EOs) extracted from Vietnamese plants were screened in vitro for the first time on *Leishmania mexicana mexicana (Lmm)* promastigotes at the maximum concentration of 50 nL/mL. Active EOs were also analyzed for cytotoxicity on mammalian cell lines (WI38, J774) and their selectivity indices (SI) were calculated. Their composition was determined by GC-MS and GC-FID. Our results indicated that EOs extracted from *Cinnamomum cassia*, *Zingiber zerumbet*, *Elsholtzia ciliata* and *Amomum aromaticum*, possessed a moderate anti-leishmanial activity, with IC_50_ values of 2.92 ± 0.08, 3.34 ± 0.34, 8.49 ± 0.32 and 9.25 ± 0.64 nL/mL respectively. However, they also showed cytotoxicity with SI < 10. The most promising EO was extracted from *Ocimum gratissimum*, displaying an IC_50_ of 4.85 ± 1.65 nL/mL and SI > 10. It contained 86.5% eugenol, which was demonstrated to be effective on *Lmm* with IC_50_ of 2.57 ± 0.57 nL/mL and not toxic on mammalian cells, explaining the observed activity.

## 1. Introduction

Cutaneous leishmaniasis (CL), the most common form of leishmaniasis, largely affects poor and developing countries in Africa, the Mediterranean Basin, the Middle East, central Asia (the Old World) and South America (the New World). The estimated number of new CL cases worldwide ranges from 0.7 million to 1.3 million annually [1].

CL is caused by protozoan parasites of the *Leishmania* (*L.*) genus. Infected female phlebotomine sand flies inject these parasites from their proboscis to human when they take their blood meals. Skin lesions, the clinical signs of CL, develop within several weeks or months after exposure. These lesions are sometimes self-healing without treatment, or become chronic. In cases of ulcers healing, they leave permanent deep scars, which often cause a serious social prejudice. Conversely, if the disease becomes chronic, there is a high risk of severe bacterial infection. CL in the New World, mainly caused by *L. mexicana*, *L. amazonensis*, *L. venezuelensis*, *L. braziliensis*, *L. guyanensis*, *L. panamensis*, and *L. peruviana*, is usually more severe and lasts longer than that in the Old World, which is caused by *L. tropica*, *L. major*, *L. aethiopica*, *L. infantum* and *L. donovani*. Moreover, CL due to *L. mexicana*, *L. amazonensis* and *L. panamensis* can develop to diffuse forms. Lesions can spread from the skin to the mucosal surfaces of the nose or the mouth, leading to chronic stuffiness, bleeding, and inflamed mucosa or sores. In advanced cases, it can lead to the ulcerative destruction of the nose (such as perforation of the nasal septum), mouth, and pharynx [1,2].

Drugs are the only therapeutic option for the treatment of CL as there is no vaccine at the moment. However, existing drugs, such as pentavalent antimonials and miltefosine, have serious drawbacks in terms of safety, efficacy, cost and difficulty in administration [3,4]. Moreover, diffuse CL initially responding to the standard treatment relapses and becomes unresponsive to further treatments [2]. Research on a safer, more effective, and shorter-course treatment for CL is therefore urgent. Nevertheless, the development of new medicines for treatment of this disease is challenging because of the variety of parasite species, pathology and immune responses [2].

Essential oils (EOs), also known as volatile oils, are natural products formed by a mixture of volatile compounds produced by many plants. They are known to be biologically active, mainly possessing antibacterial, antifungal, and antioxidant properties. There are more and more studies on EOs biological activity because they are usually devoid of long-term genotoxic risks [5]. Interestingly, topical application, the most frequent way to administer EOs, is recommended to be explored for the development of new anti-leishmanial drugs targeting CL because of their effectiveness and safety [2]. Several EOs showed interesting potential to be used as new anti-parasitic drugs such as those extracted from *Chenopodium ambrosioides* L. (a synonym of *Dysphania ambrosioides* (L.) Mosyakin & Clemant) [6,7] or *Bixa orellana* L. [8]. However, EOs are complex mixtures of several compounds and their compositions vary according to many factors, such as plant’s environment and growing conditions, methods of harvesting, extraction and storage. Moreover, the major component of an EO can also vary in different chemotypes of the same plant species. This chemical variability can influence their activities or adverse effects. Therefore, a clear knowledge of the EO composition is necessary [9].

With the aim of discovering new natural products against *Leishmania mexicana mexicana* and developing active EOs or compounds for topical application, 37 Vietnamese plants were selected for anti-leishmanial investigation in our work. It is the first time that these Vietnamese EOs were tested on this *Leishmania* species. Moreover, this study also presents the results of chemical composition analyses of the most interesting samples.

## 2. Results

A primary screening was performed at concentrations of 50 and 25 nL/mL for the 37 EOs. The results showed that among them five EOs, extracted from *Amomum aromaticum*, *Cinnamomum cassia*, *Elsholtzia ciliata*, *Ocimum gratissimum*, and *Zingiber zerumbet*, displayed a promising effect against *Leishmania mexicana mexicana* (*Lmm*) promastigotes with less than 1% viable parasites at the lower concentration tested (Table 1).

These selected EOs were further analyzed for dose-response activity to calculate IC_50_ value and for cytotoxicity on mammalian cells. The results are summarized in Table 2. All selected EOs revealed high potential against promastigote form of *Lmm* with IC_50_ values lower than 10 nL/mL. However, four of them also showed toxicity on mammalian cells, as indicated by their SI < 10. Our data demonstrated that the most active and selective EO was extracted from *Ocimum gratissimum* with an IC_50_ value on *Lmm* promastigotes of 4.85 ± 1.65 nL/mL and no toxicity towards mammalian cells at the highest tested concentration (50 nL/mL), as evidenced by more than 80% viable cells after 72 h of incubation.

To define their composition and further determine active compounds, the five selected EOs were analyzed by GC-MS and GC-FID (Table 3). Identified compounds accounted for more than 90% for each EO. Eucalyptol (55.2%), *trans*-cinnamaldehyde (83.6%), citral (neral and geranial) (40.2%), eugenol (86.5%), and zerumbone (60.3%) were characterized as the main components of EOs extracted from *A. aromaticum*, *C. cassia*, *E. ciliata*, *O. gratissimum*, and *Z. zerumbet*, respectively.

It is important to point out that eugenol is the major compound (86.5%) of the EO extracted from *O. gratissimum*, which was shown to be the most selective active sample. In order to understand the activity of this EO, in vitro anti-leishmanial activity and cytotoxicity of eugenol were studied. Interestingly, the IC_50_ value against *Lmm* promastigotes of eugenol was 2.57 ± 0.57 nL/mL (=2.72 µg/mL, 15.67 µM) and more than 80% of mammalian cells were living after 72 h when incubated at the maximal concentration used (50 nL/mL).

## 3. Discussion

This study analyzed the in vitro activity of 37 EOs extracted from Vietnamese plants against *Leishmania mexicana mexicana*. According to the classification of anti-parasitic activity from literature, extracts that showed effect against parasites with IC_50_ value ≤2 µg/mL (or 2 µM for pure compounds) are cited as having good activity, those with IC_50_ between 2 and 20 µg/mL (or micro molar for pure compounds) are considered as having a moderate activity, while those with higher IC_50_ value were considered as less interesting [11]. In this study, the primary screening was performed against *Lmm* promastigotes at concentrations of 25 and 50 nL/mL in order to quickly identify interesting EOs (1 nL is a little less than 1 µg depending on the density of the EO) [11]. Our data showed that percentages of viable parasites incubated with the lower concentration of five EOs, extracted from *A. aromaticum*, *C. cassia*, *E. ciliata*, *O. gratissimum*, and *Z. zerumbet*, were lower than 1%, meaning that these EOs revealed a potential activity. Indeed, the IC_50_ values of these selected EOs were lower than 10 nL/mL in the second analysis.

To have a general look at anti-leishmanial activity and cytotoxicity of the five more active EOs and to understand their observed effects, we compiled literature data of EOs extracted from similar plant species and also of their major components in Table 4.

As shown in this table, among the five selected samples, EOs obtained from *A. aromaticum*, *C. cassia*, and *E. ciliata* were analyzed here for the first time for their leishmanicidal effect. In our experiment, they showed IC_50_ values of 9.25 ± 0.64, 2.29 ± 0.08, and 8.49 ± 0.32 nL/mL, respectively against *Lmm* promastigotes. Unfortunately, this activity was not very selective on *Leishmania* as SI values compared to non-cancer mammalian cells (WI38) and cancer cells (J774) were approximately 5 and 2, respectively.

Two EOs extracted from *O. gratissimum* and *Z. zerumbet* were already investigated for anti-leishmanial activity [13,14,15,16] however on other *L.* species. The EO extracted from fresh leaves of *O. gratissimum* collected in Brazil did not reveal interesting effects against both *L. amazonensis* and *L. chagasi*. On the contrary, in our study, the EO extracted from *O. gratissimum* collected in Vietnam demonstrated notable activity against *Lmm* promastigotes, with an IC_50_ value of 4.85 ± 1.65 nL/mL. These results indicated a specific activity of this EO related to parasite species (*Leishmania mexicana mexicana*) and/or collection place and composition. Another important feature of this EO is its absence of cytotoxicity as the percentages of viable WI38 and J774 cells at the maximal tested concentration (50 nL/mL) were higher than 80%. We therefore selected the EO extracted from *O. gratissimum* as the most active and selective sample. Regarding the EO extracted from fresh rhizomes of *Z. zerumbet*, its activity against *L. donovani* was in the same range than what we found against *Lmm* promastigotes (IC_50_ = 3.34 ± 0.34 nL/mL). Unfortunately, we also observed cytotoxicity of this EO on WI38 and J774 cells as shown by SI values of 1.10 and 0.72, respectively.

Eucalyptol, the major compound of the EO extracted from *A. aromaticum* (55.2%), was not active against the different tested *L.* species [17,18]. Other components present in our *A. aromaticum* EO, such as citral (neral and geranial, 9.6%), 2-(*E*)-decenal (5.3%), α-terpineol (2.8%), β-pinene (2.6%) and α-pinene (2.1%) may be therefore responsible, in part, of the observed activity. Among these compounds, 2-(*E*)-decenal and α-pinene have already shown leishmanicidal effects. IC_50_ values of 2-(*E*)-decenal against *L. donovani* promastigotes and axenic amastigotes were 7.85 ± 0.28 and 2.47 ± 0.25 µg/mL respectively [29], and IC_50_ value of α-pinene against *L. amazonensis* promastigotes, *L. amazonensis* axenic amastigotes and *L. infantum* promastigotes were 19.7, 16.1 and 45.94 µg/mL respectively [17,30].

Citral was characterized as the main component of the EO extracted from *E. ciliata* (40.2%). It showed a moderate activity against *L. donovani*, *L. amazonensis* and a weak effect against *L. infantum*, *L. tropica* and *L. major* [20,21,22]*.* However, it is important to point out that this compound is not selective on *Leishmania* as shown by the toxicity on kidney epithelial and J774 cells [20,21]. Our results indicated activity on parasites and mammalian cells of the EO extracted from *E. ciliata* in the same range than citral with IC_50_ value of 8.49 ± 0.32 nL/mL and SI of 5.58 (compared to WI38) and 1.56 (compared to J774). Nevertheless, citral being present at only 40.2% in the *E. ciliata* EO, other compounds should also have anti-leishmanial activity as β-(*E*)-ocimene (14.0%), linalool (8.3%), 1-octen-3-ol (7.1%) and β*-*(*E*)-farnesene (6.2%). Dutra et al. have already examined leishmanicidal effect of linalool on *L. infantum chagasi*. However when axenic amastigotes were treated with linalool, IC_50_ was 550 µg/mL [24].

EO extracted from *C. cassia* contained 83.6% of *trans*-cinnamaldehyde. The anti-leishmanial activity of this compound is unknown but its toxicity on two human cell lines was determined [19]. In our sample, the high percentage of cinnamaldehyde may explain the toxicity of this EO on both mammalian cell lines, WI38 and J774, with IC_50_ of 14.19 ± 0.54 and 6.26 ± 0.80 nL/mL respectively. Given the known cytotoxicity of this compound and its high concentration in the *C. cassia* EO, we did not find interesting to further analyze its anti-parasitic activity.

As mentioned previously, eugenol was characterized as the major compound (86.5%) of the most interesting EO extracted from our Vietnamese sample of *O. gratissimum*. Literature data indicated a moderate effect on *L. amazonensis* and a less interesting activity against *L. infantum chagasi* of eugenol [23,24,25]. On L. *donovani*, along with a moderate in vitro activity, in vivo effect of eugenol (emulsified) was determined. The intra-peritoneal administration of an eugenol emulsion at the dose of 75 mg/kg b.w. for 10 consecutive days decreased by 87.01 ± 5.85 and 86.68 ± 5.42% parasitic load in spleen and liver, respectively, in 8-weeks infected BALB/c mice. Moreover, a significant reduction in spleen size, and spleen and liver weights were also found at this dose [26]. Our results further support the anti-leishmanial activity of this compound with IC_50_ value of 2.57 ± 0.57 nL/mL (=2.72 µg/mL or 15.67 µM) against *Lmm* promastigotes. Interestingly, eugenol was not toxic on both non-cancer and cancer mammalian cells in our models at the highest analyzed concentration (50 nL/mL). From these results, and its high percentage in the *O. gratissimum* EO, it can be concluded that activity of eugenol can explain the anti-leishmanial activity of this EO.

Zerumbone, accounting for 60.3% of the EO extracted from fresh rhizomes of *Z. zerumbet*, was reported to be active against *L. donovani*, however it revealed toxicity on human leukemia cells HL-60 [27,28]. These data can explain the anti-leishmanial activity but also cytotoxicity of the *Z. zerumbet* EO found in our study.

Mechanisms of anti-leishmanial activity of these EOs and their major compounds are not well known. Usually, effects were analyzed on the morphology of treated parasites or as a consequence of immunostimulatory activities.

Most of the studies used transmission electron microscopy (TEM) and scanning electron microscopy (SEM) to analyze the morphology of EOs treated parasites. The EO extracted from *O. gratissimum* caused considerable mitochondrial swelling in *L. amazonensis* promastigotes at 135 µg/mL and amastigotes at 100 µg/mL [13], swelling of cell body, flagellar pocket and mitochondria in *L. chagasi* promastigotes at 50 µg/mL [15]. Ultrastructure alterations were also observed in *L. amazonenesis* and *L. infantum* promastigotes treated with citral at concentrations of 8.0 and 42 µg/mL respectively [21,22]. Mukherjee et al. detected morphological alterations by SEM in *L. donovani* promastigotes treated with 9.36 µM of zerumbone associated with induced ROS-mediated apoptosis [27].

Using other experiments, citral at the concentration of 42 µg/mL was shown to trigger programmed cell death of *L. infantum* promastigotes, as indicated by the externalization of phosphatidylserine, loss of mitochondrial membrane potential and cell-cycle arrest at the G(0)/G(1) phase [22].

Regarding immunostimulatory activity, an increase of nitric oxide produced by infected macrophages has been suggested to be responsible for the activity of the *O. gratissimum* EO against *L. amazonensis* at 100 and 150 µg/mL [13]. Islamuddin et al. explored the synergic effect between eugenol emulsion and the immune system. In BALB/mice infected by *L. donovani*, treatment with 75 mg/kg b.w. of eugenol emulsion enhanced IFN-γ and IL-2 serum levels, as well as increased CD4+ and CD8+ T cell population and expanded IFN-γ producing CD4+ and CD8+ splenic T lymphocytes [26].

Concerning the mechanism of eugenol activity, although no data are available on *Lmm* promastigotes, some experiments were carried out on bacteria and fungi. Eugenol at concentrations of 5.3 and 10.6 mg/mL was reported to significantly damage both the cell wall and membrane of the treated Gram-negative and Gram-positive bacteria [31]. Khan et al. observed damaging effects of eugenol at 200 µg/mL on cell wall, cell membrane, cytoplasmic contents and other membranous structures of treated *Candida albicans* [32]. Indeed, because of the lipophilicity of eugenol, it could easily diffuse between the fatty acyl chains of lipid bilayers modifying the fluidity, integrity and permeability of cell membranes [31]. We now intend to analyze possible effects of this compound on leishmanial membranes.

## 4. Materials and Methods

### 4.1. Plants Collection

Thirty-seven plants were collected from different areas of Vietnam in November 2014 and from May to August 2015. They were identified by matching with literature and herbarium specimen at the Botanical Department, Hanoi University of Pharmacy, Vietnam. The information of sample name, genus, species, family, and collector name is given in the supplementary material.

### 4.2. Essential Oils Extraction

Fresh samples were extracted by hydro-distillation using a modified Clavenger apparatus for two hours. Each essential oil obtained from hydro-distillation was dried using sodium sulfate, filtered and kept refrigerated prior to analysis. Stock solutions at the concentration of 20 µL/mL were prepared in DMSO and diluted further in culture medium to achieve a maximum final DMSO concentration of 0.25%.

### 4.3. Culture Maintenance

Promastigote form of *Leishmania mexicana mexicana* (*Lmm*, MHOM/BZ/84/BEL46) was grown in SDM 79 medium (Life technologies, Ghent, Belgium) supplemented with 15% heat-inactivated fetal bovine serum and 0.2% hemin. The culture was maintained at 28 °C in 5% CO_2_ incubator. The human non-cancer fibroblast cell line WI38 (ATCC Number CCL-75 from LGC Standards, Middlesex, UK) and macrophage-like murine cell line J774 (ECACC Number 91051511 from Public Health England, Salisbury, UK) were grown in DMEM and RPMI medium (Gibco from Thermo Fisher Scientific, Merelbeke, Belgium or Sigma-Aldrich, Bornem, Belgium), respectively, supplemented with 10% fetal bovine serum and penicillin-streptomycin (100 UI/mL). The culture was maintained at 37 °C in 5% CO_2_ incubator.

### 4.4. Anti-leishmanial Assay

The *Lmm* promastigote density was counted on a haemocytometer and adjusted to 10^5^ parasites/mL. The assays were performed in 96-well plates. For primary screening (repeated two times at concentrations of 50 and 25 nL/mL in triplicate) essential oils were diluted in culture medium from the stock solutions (20 µL/mL). Each well was filled with 50 μL of the diluted essential oil and 50 μL of the *Lmm* promastigote culture (total volume 100 μL). After 72 h of incubation, 10 µL Alamar blue (Thermo Fisher Scientific, diluted with PBS at the ratio 1:1) was added to each well and the plates were further incubated for 4 h. Fluorescence was measured on a spectrophotometer (SpectraMax-Molecular Devices, Berkshire, UK) at 530 nm excitation and 590 nm emission wavelengths. Pentamidine was tested as standard drug. The essential oils that inhibited more than 50% the growth of *Lmm* promastigotes at the concentration of 25 nL/mL in the primary screening were submitted to a second analysis for accurate IC_50_ determination. Selected essential oils were screened at least three times at concentrations ranging from 50 to 0.02 nL/mL in duplicate. IC_50_ values were calculated from dose response growth inhibition curves by Microsoft excel files.

### 4.5. Cytotoxicity Assay

The essential oils which were analyzed for IC_50_ value on anti-leishmanial assay were also analyzed for cytotoxicity against non-cancer (WI38) and cancer (J774) mammalian cells. The culture of cells was diluted with medium to the adequate density of 5 × 10^3^ cells/mL and then 180 μL was added into each well of 96-well plates. After 24 h of incubation, 20 μL of essential oils diluted in culture medium was added to each well (to obtain concentrations ranging from 50−0.02 nL/mL) for further 72 h of incubation. After removing the medium, 100 μL of MTT was added to each well and the plates were further incubated for 45 minutes. 100 μL of DMSO was used to dissolve formed formazan crystals after MTT removing. Absorbance was measured on a spectrophotometer (SpectraMax-Molecular Devices, Berkshire, UK) at 570 nm with a reference wavelength at 620 nm. All experiments were made at least two times in triplicate. The computation of the IC_50_ values was performed with GraphPad Prism 5.0 (GraphPad Software Inc., San Diego, CA, USA). The selectivity index (SI) values were calculated using the formula below:

SI = IC_50_ for mammalian cell/IC_50_ for protozoan parasite

### 4.6. Essential Oil Analysis

The GC-MS analyses were carried out on a TRACE GC 2000 series (Thermo-Quest, Rodano, Italy), with a DB-WAX capillary column (30 m × 0.25 mm × 0.25 µm) using the following operating conditions: injection volume: 1 µL (TBME solution); injection mode: splitless; injector temperature: 230 °C; oven temperature: increased from 45 °C (held on 5 min) to 250 °C (held on 5 min) at 3 °C/min; helium was used as a carrier gas at a constant flow of 1.3 mL/min; detector temperatures: 260 °C; ion source: 70 eV. The oil components were identified using linear retention indices in relation to a series of fatty acid methyl esters (C_5_–C_27_), pure compounds and NIST mass spectral library (matching coefficient > 700). GC-FID was done using the same column and conditions on a FOCUS GC (Thermo Finnigan, Milan, Italy) with modifications of injection mode (split at ratio 1:50) and detector temperature (250 °C). Percentage of compounds was calculated by the normalization procedure.

## 5. Conclusions

The present study analyzes for the first time the anti-leishmanial activity of 37 essential oils extracted from Vietnamese plants. Those extracted from *A. aromaticum*, *C. cassia* and *E. ciliata* are shown here for the first time to be effective on a *Leishmania* species, while the effects of *O. gratissimum* and *Z. zerumbet* EOs are reported for the first time on *L. mexicana* species. More than 90% of their contents was characterized. Eugenol was identified as the major compound of the *O. gratissimum* EO, the most active and selective one. This compound, showing a moderate anti-leishmanial activity and low cytotoxicity in the tested models, can explain this EO activity. However, further results are necessary before developing it in the treatment of cutaneous leishmaniasis such as in vivo assessment in *Lmm* infected animals and determination of its mode of action.

## Figures and Tables

**Table 1:** Viability of *Lmm* promastigotes in the presence of 25 nL/mL EO.

Plant Species	EO Obtained From	Viability of *Lmm* promastigotes (%) (Average ± Standard Deviation)
*Ageratum conyzoides* (L.) L.	leaves	89.84 ± 1.58
*Alpinia galanga* (L.) Willd.	rhizomes	93.02 ± 4.90
***Amomum aromaticum* Roxb.**	**fruits**	**0.27 ± 0.05**
*Amomum schmidtii* (K. Schum.) Gagnep.	rhizomes	96.82 ± 1.13
*Anethum graveolens* L.	fruits	96.53 ± 1.26
*Artemisia annua* L.	leaves	92.32 ± 1.55
*Blumea lanceolaria* (Roxb.) Druce	leaves	93.59 ± 1.24
***Cinnamomum cassia* (L.) J. Presl**	**stem barks**	**0.48 ± 0.01**
*Clausena indica* (Dalzell) Oliv.	leaves	95.07 ± 7.40
*Coriandrum sativum* L.	fruits	94.47 ± 2.38
*Curcuma longa* L.	rhizomes	92.90 ± 1.61
*Curcuma zedoaria* (Christm.) Roscoe	rhizomes	99.53 ± 0.23
*Dysphania ambrosioides* (L.) Mosyakin & Clemant	leaves, fruits	92.69 ± 1.93
*Elsholtzia blanda* (Benth.) Benth.	leaves	94.70 ± 6.12
***Elsholtzia ciliata* (Thunb.) Hyl.**	**leaves**	**0.38 ± 0.00**
*Elsholtzia communis* (Collett & Hemsl.) Diels	leaves	97.24 ± 2.19
*Elsholtzia penduliflora* W. W. Sm.	leaves	97.88 ± 0.37
*Eucalyptus camaldulensis* Dehnh.	leaves	101.13 ± 0.54
*Hedychium coronarium* J.Koenig	rhizomes	91.79 ± 4.60
*Hyptis suaveolens* (L.) Poit.	leaves	67.32 ± 1.97
*Illicium verum* Hook. f.	fruits	93.35 ± 5.29
*Kaempferia galanga* L.	rhizomes	100.00 ± 7.47
*Litsea cubeba* (Lour.) Pers.	fruits	96.00 ± 5.18
*Litsea cubeba* (Lour.) Pers.	leaves	94.67 ± 2.99
*Melaleuca alternifolia* (Maiden & Betch) Cheel	leaves	87.61 ± 4.93
*Melaleuca cajuputi* Powell	leaves	92.53 ± 3.17
***Ocimum gratissimum* L.**	**leaves**	**0.13 ± 0.01**
*Ocimum tenuiflorum* L.	leaves	89.08 ± 5.23
*Piper sarmentosum* Roxb.	leaves	81.43 ± 14.12
*Platycladus orientalis* (L.) Franco	leaves	92.41 ± 16.49
*Plectranthus amboinicus* (Lour.) Spreng.	leaves	82.06 ± 1.50
*Pluchea indica* (L.) Less.	leaves	88.07 ± 4.37
*Pogostemon cablin* (Blanco) Benth.	leaves	93.64 ± 1.51
*Vitex trifolia* L.	leaves	88.70 ± 3.34
*Zingiber montanum* (J.Koenig) Link ex A.Dietr.	rhizomes	88.36 ± 1.59
*Zingiber officinale* Roscoe	rhizomes	94.29 ± 3.83
***Zingiber zerumbet* (L.) Roscoe ex Sm.**	**rhizomes**	**0.19 ± 0.05**

**Table 2:** In vitro anti-leishmanial activity, cytotoxicity and selectivity indices of the five selected EOs.

Plant Species	Anti-Leishmanial Activity (IC_50_ nL/mL) Average ± Standard Deviation	Cytotoxicity (IC_50_ nL/mL) Average ± Standard Deviation			
		WI38	SI (WI38)	J774	SI (J774)
*Amomum aromaticum*	9.25 ± 0.64	47.31 ± 0.30	5.11	22.68 ± 3.22	2.45
*Cinnamomum cassia*	2.92 ± 0.08	14.19 ± 0.54	4.85	6.26 ± 0.80	2.14
*Elsholtzia ciliata*	8.49 ± 0.32	47.38 ± 1.64	5.58	13.21 ± 1.48	1.56
*Ocimum gratissimum*	4.85 ± 1.65	>50	>10.3	>50	>10.3
*Zingiber zerumbet*	3.34 ± 0.34	3.68 ± 0.34	1.10	2.41 ± 0.04	0.72
Pentamidine	0.04 ± 0.006 *				
Camptothecin		0.13 ± 0.02 *		0.01 ± 0.01 *	

* concentration in µg/mL.

**Table 3:** Chemical composition of the five selected EOs.

No.	Compounds	RI	Relative Percentages (%)					Identification
			*A. aromaticum*	*C. cassia*	*E. ciliata*	*O. gratissimum*	*Z. zerumbet*	
1	α-Pinene	535	2.1	-	-	-	2.3	MS, [10], Co-GC
2	Camphene	573−576	t	-	-	-	8.0	MS, [10]
3	β-Pinene	619−621	2.6	-	0.1	-	0.2	MS, [10], Co-GC
4	Sabinene	634−635	t	-	t	0.2	-	MS, [10]
5	3-Carene	664−665	0.1	-	-	-	1.1	MS
6	α-Phellandrene	682	-	-	-	-	0.1	MS, Co-GC
7	Myrcene	684	-	-	0.2	0.2	0.4	MS, [10]
8	α-Terpinene	697	-	-	-	t	-	MS, [10], Co-GC
9	Limonene	719−720	0.8	-	4.1	-	1.0	MS, [10], Co-GC
**10**	**Eucalyptol**	**730-740**	**55.2**	**-**	**-**	**-**	1.6	MS, [10], Co-GC
11	(*Z*)-β-Ocimene	758−760	-	-	0.7	5.4	-	MS
12	γ-Terpinene	765−766	-	-	t	0.1	t	MS, [10], Co-GC
13	(*E*)-β-Ocimene	773−778	-	-	14.0	0.2	-	MS
14	*p*-Cymene	787−792	0.6	t	t	t	0.2	MS, [10], Co-GC
15	Terpinolene	800−801	-	-	t	t	0.1	MS, [10], Co-GC
16	Octanal	812	0.2	-	-	-	-	MS
17	(*E*)-3-Hexen-1-ol acetate	837	-	-	t	-	-	MS
18	5-Hepten-2-one, 6-methyl-	855−858	0.2	-	1.1	-	-	MS, Co-GC
19	α-Pinene oxide	875	-	-	0.1	-	-	MS
20	*allo*-Ocimene	887	-	-	-	0.1	-	MS, Co-GC
21	1-Octen-1-yl acetate	897	-	-	1.0	-	-	MS
22	(*Z*)-3-Hexen-1-ol	902−904	-	-	0.5	t	-	MS, Co-GC
23	Fenchone	907	-	-	-	-	0.1	MS, Co-GC
24	3-Octanol	915	-	-	0.3	-	-	MS, [10]
25	(*E*)-2-Octenal	945	0.6	-	-	-	-	MS
26	*cis*-Linalool oxide (furanoid)	961	t	-	-	-	-	MS, Co-GC
27	1-Octen-3-ol	971	-	-	7.1	-	-	MS, [10]
28	*cis*-Sabinene hydrate	980	-	-	-	0.3	-	MS
29	Cyclosativene	987	-	0.1	-	-	-	MS
30	*trans-*Linalool oxide (furanoid)	988	t	-	-	-	-	MS, Co-GC
31	Citronellal	990	-	-	t	-	-	MS, [10], Co-GC
32	α-Copaene	998−1003	-	4.2	-	0.2	-	MS
33	Camphor	1020−1021	-	-	0.4	-	2.1	MS, [10], Co-GC
34	β-Bourbonene	1023	-	-	-	0.2	-	MS
35	Benzaldehyde	1027	-	1.0	t	-	-	MS
36	β-Cubebene	1044	-	-	-	0.1	-	MS
37	Linalool	1060−1065	0.7	-	8.3	0.1	0.3	MS, [10], Co-GC
38	Terpinen-1-ol	1077	0.1	-	-	-	-	MS
39	Bornyl acetate	1086	-	-	-	-	0.1	MS, Co-GC
40	*trans-*α-Bergamotene	1091−1092	-	t	t	-	-	MS, [10]
41	β-Elemene	1093−1094	-	0.2	-	0.1	-	MS
42	β-Caryophyllene	1098−1101	-	0.1	3.0	1.3	0.7	MS, [10], Co-GC
43	Terpinen-4-ol	1108−1113	0.9	t	t	0.2	0.2	MS, [10], Co-GC
44	Acetophenone	1149	-	-	0.7	-	-	MS
45	(*E*)-2- Decenal	1153	5.3	-	-	-	-	MS
46	α-Humulene	1166−1173	-	t	t	t	9.2	MS, [10], Co-GC
47	(*E*)-β-Farnesene	1178	-	-	6.2	-	-	MS
48	γ-Muurolene	1187−1188	-	0.5	-	t	-	MS
**49**	**Neral**	1187−1188	3.2	-	**16.8**	-	-	MS, Co-GC
50	Methyl geranate	1197	-	-	0.2	-	-	MS
51	α-Terpineol	1201−1207	2.8	-	0.4	-	0.2	MS, [10], Co-GC
52	Borneol	1205	-	-	-	-	0.2	MS, [10], Co-GC
53	Germacrene D	1207	-	-	-	3.4	-	MS, [10]
54	Geranyl formate	1212	0.6	-	-	-	-	MS
55	β-Selinene	1214	-	0.1	-	-	-	MS
56	Neryl acetate	1218	-	-	t	-	-	MS, [10], Co-GC
57	α-Muurolene	1224	-	0.8	-	-	-	MS
**58**	**Geranial**	1238	6.4	-	**23.4**	-	-	MS
59	β-Cadinene	1253−1254	-	-	-	0.2	0.1	MS
60	δ-Cadinene	1257	-	2.0	-	-	-	MS
61	Geranyl acetate	1259−1263	0.9	-	0.7	-	-	MS, [10], Co-GC
62	Citronellol	1272	-	-	0.5	-	-	MS, [10], Co-GC
63	Benzenepropanal	1273	-	1.7	-	-	-	MS
64	*cis-*Cadina-1,4-diene	1278	-	0.5	-	-	-	MS
65	Isogeraniol	1289	-	-	0.2	-	-	MS
66	Nerol	1304	-	-	3.5	-	-	MS, [10], Co-GC
67	*cis*-Isogeraniol	1311	-	-	0.4	-	-	MS
68	(*E*)-2,6-dimethyl-3,5,7-octatriene-2-ol	1323−1324	-	-	t	0.1	-	MS
69	*cis*-Calamenene	1325	-	0.5	-	-	-	MS
70	Geraniol	1350−1354	2.4	-	3.1	-	-	MS, [10], Co-GC
71	(*E*)-2-Dodecenal	1359	1.5	-	-	-	-	MS
72	*cis*-Cinnamaldehyde	1382	-	2.4	-	-	-	MS
73	Indane-4-carboxalde-hyde	1466	1.7	-	-	-	-	MS
74	Caryophyllene oxide	1466−1501	-	-	0.6	0.2	3.6	MS, [10], Co-GC
75	Humulene epoxide II	1522	-	-	-	-	2.2	MS
**76**	***trans-*Cinnamaldehyde**	1534	-	**83.6**	-	-	-	MS, Co-GC
77	(*E*)-Nerolidol	1533−1538	1.2	-	0.5	-	-	MS, [10], Co-GC
78	Cubenol	1552	-	0.2	-	-	-	MS
79	Elemol	1571	0.2	-	-	-	-	MS
80	Cinnamyl acetate	1633	-	0.9	-	-	-	MS
**81**	**Eugenol**	1657	-	-	-	**86.5**	-	MS, Co-GC
82	α-Muurolol	1669	-	0.1	-	-	-	MS
83	β-Eudesmol	1706	-	-	-	-	0.2	MS
84	α-Cadinol	1710	-	-	-	0.1	-	MS
85	Cinnamyl alcohol	1761	-	0.2	-	-	-	MS, Co-GC
**86**	**Zerumbone**	1831	-	-	-	-	60.3	MS
87	*o*-Methoxy-cinnamaldehyde	1902	-	0.3	-	-	-	MS
88	*trans*-Phytol	2085	-	-	0.16	-	-	MS, Co-GC
	**Total identified**		**90.3**	**99.4**	**98.3**	**99.2**	**94.5**	

t: trace (peak area less than 0.05%); RI: the retention index was calculated using a homologous series of fatty acid methyl esters C_5_–C_27_; MS: mass spectra (matching coefficient >700 compared with NIST database); Co-GC: co-injection with pure compound.

**Table 4:** Profile of in vitro anti-leishmanial activity and cytotoxicity of the five more active EOs and their major compounds.

Plants/Compounds	Anti-Leishmanial Activity		Cytotoxicity		Refs.
	*L.* Species-Form	IC_50_ (µg/mL)	Cell Line	IC_50_ (µg/mL)	
*A. aromaticum*	ND		ND		
*C. cassia*	ND		ND		
*E. ciliata*	ND		PC12 rat pheochromocytoma cells	>50	[12]
*O. gratissimum*	*L. amazonensis*-proma.	135	CHO	125.00 ± 1.68	[13,14]
	*L. amazonensis*-ama.	100	WI38	165.51 ± 6.81	[13,14]
	*L. chagasi*-proma.	80			[15]
*Z. zerumbet*	*L. donovani*-proma.	4.62	ND		[16]
Eucalyptol	*L. infantum*-proma.	>100	Vero cell	63.49	[17]
	*L. infantum*-ama.	>100			[17]
	*L. infantum*-proma.	>400			[18]
	*L. tropica*-proma.	>400			[18]
	*L. major*-proma.	>400			[18]
Cinnamal-dehyde	ND		human embryonic stem cells	4.88	[19]
			human pulmonary fibroblasts	5.28	[19]
Citral	*L. donovani*-proma.	19	kidney epithelial cell	22.4	[20]
	*L. amazonensis*-proma.	8.0 ± 0.06	J774	50.0 ± 0.10	[21]
	*L. amazonensis*-ama.	25 ± 0.29			[21]
	*L. infantum*-proma.	42			[22]
	*L. tropica*-proma.	34			[22]
	*L. major*-proma.	36			[22]
Eugenol	*L. amazonensis*-proma.	12.65	red blood cell	>65.6	[23]
	*L. infantum chagasi*-proma.	500	BALB/c peritoneal macrophages	300	[24]
	*L. infantum chagasi*-axe. ama.	220			[24]
	*L. infantum chagasi* infected macrophages	100			[24]
	*L. infantum chagasi*-proma.	56.13 ± 2.09			[25]
	*L. infantum chagasi*-ama.	20.81 ± 1.59			[25]
Eugenol (emulsified)	*L. donovani*-proma.	8.43 ± 0.96	murine macrophages	>200	[26]
	*L. donovani*-ama.	5.05 ± 1.72			[26]
Zerumbone	*L. donovani*-proma.	2.04	HL-60	2.27	[27,28]

ND: not determined; proma.: promastigote; ama.: intracellular amastigote; axe. ama.: axenic amastigote.

## Supplementary Materials

Supplementary materials are available online.
