# Supplementary material for: In Vitro Anti-Leishmanial Activity of Essential Oils Extracted from Vietnamese Plants
Source: Molecules. 2017 Jun 27;22(7):1071. doi: 10.3390/molecules22071071 (PMC6152080; doi:10.3390/molecules22071071)
Supplement: Supplementary file 1 [file molecules-22-01071-s001.zip › Supplementary materials/Certificate of number sign of samples.pdf]

HANOI UNIVERSITY OF PHARMACY, VIETNAM

DEPARTMENT OF BOTANY

HERBARIUM OF MEDICINAL PLANTS (HNIP)

****

CERTIFICATE OF NUMBER SIGN OF SAMPLES

No.	Sign	Collector & Sender	Date of collection	Date sent	Scientific name	Vietnamese name	Locality	Determined by	Duplicate	Number sign in HNIP
1	TD_01	Le Thanh Binh	05/2015	08/08/2016	*Melaleuca alternifolia* (Maiden & Betch) Cheel	Tràm Úc	Hà Nội	Msc. Nghiem Duc Trong	01	HNIP/18250/16
2	TD_02	Le Thanh Binh	05/2015	08/08/2016	*Ocimum gratissimum* L.	Hương nhu trắng	Hà Nội	Msc. Nghiem Duc Trong	01	HNIP/18251/16
3	TD_03	Le Thanh Binh	05/2015	08/08/2016	*Pluchea indica* (L.) Less.	Cúc tần	Hà Nội	Msc. Nghiem Duc Trong	01	HNIP/18252/16
4	TD_04	Le Thanh Binh	05/2015	08/08/2016	*Platycladus orientalis* (L.) Franco	Trắc bách	Hà Nội	Msc. Nghiem Duc Trong	01	HNIP/18253/16
5	TD_05	Le Thanh Binh	06/2015	08/08/2016	*Piper sarmentosum* Roxb.	Lá lốt	Hà Nội	Msc. Nghiem Duc Trong	01	HNIP/18254/16
6	TD_06	Le Thanh Binh	08/2015	08/08/2016	*Artemisia annua* L.	Thanh hao hoa vàng	Hà Nội	Msc. Nghiem Duc Trong	01	HNIP/18255/16
7	TD_07	Le Thanh Binh	05/2015	08/08/2016	*Ocimum tenuiflorum* L.	Hương nhu tía	Hà Nội	Msc. Nghiem Duc Trong	01	HNIP/18256/16
8	TD_08	Le Thanh Binh	08/2015	08/08/2016	*Dysphania ambrosioides* (L.) Mosyakin & Clemant	Dầu giun	Thanh Hoá	Msc. Nghiem Duc Trong	01	HNIP/18257/16
9	TD_09	Le Thanh Binh	05/2015	08/08/2016	*Blumea lanceolaria* (Roxb.) Druce	Xương sông	Thanh Hoá	Msc. Nghiem Duc Trong	01	HNIP/18258/16
10	TD_10	Le Thanh Binh	05/2015	08/08/2016	*Eucalyptus camaldulensis* Dehnh.	Bạch đàn trắng	Hà Nội	Msc. Nghiem Duc Trong	01	HNIP/18259/16
11	TD_11	Le Thanh Binh	2015	08/08/2016	*Litsea cubeba* (Lour.) Pers.	Màng tang	Quảng Nam	Msc. Nghiem Duc Trong	01	HNIP/18260/16

No.	Sign	Collector & Sender	Date of collection	Date sent	Scientific name	Vietnamese name	Locality	Determined by	Duplicate	Number sign in HNIP
12	TD_12	Le Thanh Binh	06/2015	08/08/2016	*Kaempferia galanga* L.	Địa liền	Hà Nội	Msc. Nghiem Duc Trong	01	HNIP/18261/16
13	TD_13	Le Thanh Binh	07/2015	08/08/2016	*Zingiber zerumbet* (L.) Roscoe ex Sm.	Gừng gió	Hà Nội	Msc. Nghiem Duc Trong	01	HNIP/18262/16
14	TD_14	Le Thanh Binh	08/2015	08/08/2016	*Ageratum conyzoides* (L.) L.	Cây cứt lợn	Thanh Hoá	Msc. Nghiem Duc Trong	01	HNIP/18263/16
15	TD_18	Le Thanh Binh	07/2015	08/08/2016	*Curcuma longa* L.	Nghệ	Hà Nội	Msc. Nghiem Duc Trong	01	HNIP/18264/16
16	TD_19	Le Thanh Binh	07/2015	08/08/2016	*Pogostemon cablin* (Blanco) Benth.	Hoắc hương	Hưng Yên	Msc. Nghiem Duc Trong	01	HNIP/18265/16
17	TD_20	Le Thanh Binh	07/2015	08/08/2016	*Hyptis suaveolens* (L.) Poit.	É lớn tròng	Vĩnh Phúc	Msc. Nghiem Duc Trong	01	HNIP/18266/16
18	TD_22	Le Thanh Binh	05/2015	08/08/2016	*Vitex trifolia* L.	Mạn kinh	Hà Nội	Msc. Nghiem Duc Trong	01	HNIP/18267/16
19	TD_23	Le Thanh Binh	08/2015	08/08/2016	*Clausena indica* (Dalzell) Oliv.	Mác mật	Thái Nguyên	Msc. Nghiem Duc Trong	01	HNIP/18268/16
20	TD_24	Le Thanh Binh	08/2015	08/08/2016	*Elsholtzia blanda* (Benth.) Benth.	Kinh giới núi	Lào Cai	Msc. Nghiem Duc Trong	01	HNIP/18269/16
21	TD_25	Le Thanh Binh	08/2015	08/08/2016	*Elsholtzia penduliflora* W. W. Sm.	Chùa dù	Lào Cai	Msc. Nghiem Duc Trong	01	HNIP/18270/16
22	TD_26	Le Thanh Binh	08/2015	08/08/2016	*Litsea cubeba* (Lour.) Pers.	Màng tang	Lào Cai	Msc. Nghiem Duc Trong	01	HNIP/18271/16
23	TD_27	Le Thanh Binh	2015	08/08/2016	*Illicium verum* Hook. f.	Hồi	Lạng Sơn	Msc. Nghiem Duc Trong	01	HNIP/18272/16
24	TD_28	Le Thanh Binh	07/2015	08/08/2016	*Zingiber officinale* Roscoe	Gừng	Hà Nội	Msc. Nghiem Duc Trong	01	HNIP/18273/16
25	TD_29	Le Thanh Binh	08/2015	08/08/2016	*Hedychium coronarium* J.Koenig	Ngải tiên trắng, Bạch yến	Hà Nội	Msc. Nghiem Duc Trong	01	HNIP/18274/16
26	TD_30	Le Thanh Binh	07/2015	08/08/2016	*Coriandrum sativum* L.	Mùi	Hà Nội	Msc. Nghiem Duc Trong	01	HNIP/18275/16
27	TD_32	Le Thanh Binh	07/2015	08/08/2016	*Anethum graveolens* L.	Thì là	Hà Nội	Msc. Nghiem Duc Trong	01	HNIP/18276/16

No.	Sign	Collector & Sender	Date of collection	Date sent	Scientific name	Vietnamese name	Locality	Determined by	Duplicate	Number sign in HNIP
28	TD_33	Le Thanh Binh	07/2015	08/08/2016	*Plectranthus amboinicus* (Lour.) Spreng.	Húng chanh	Phú Thọ	Msc. Nghiem Duc Trong	01	HNIP/18277/16
29	TD_34	Le Thanh Binh	08/2015	08/08/2016	*Elsholtzia ciliata* (Thunb.) Hyl. *(Syn. = Elsholtzia cristata* Willd.)	Kinh giới	Hà Nội	Msc. Nghiem Duc Trong	01	HNIP/18278/16
30	TD_35	Le Thanh Binh	08/2015	08/08/2016	*Zingiber montanum* (J.Koenig) Link ex A.Dietr.	Gừng núi	Hà Nội	Msc. Nghiem Duc Trong	01	HNIP/18279/16
31	TD_36	Le Thanh Binh	08/2015	08/08/2016	*Amomum schmidtii* (K.Schum.) Gagnep.	Sa nhân hồi	Quảng Ninh	Msc. Nghiem Duc Trong	01	HNIP/18280/16
32	TD_38	Le Thanh Binh	11/2014	08/08/2016	*Elsholtzia communis* (Collett & Hemsl.) Diels	Kinh giới Đồng Văn	Hà Giang	Msc. Nghiem Duc Trong	01	HNIP/18281/16
33	TD_39	Le Thanh Binh	08/2015	08/08/2016	*Amomum aromaticum* Roxb.	Thảo quả	Lào Cai	Msc. Nghiem Duc Trong	01	HNIP/18282/16
34	TD_40	Le Thanh Binh	08/2015	08/08/2016	*Cinnamomum cassia* (L.) J.Presl	Quế	Quảng Ninh	Msc. Nghiem Duc Trong	01	HNIP/18283/16
35	TD_42	Le Thanh Binh	08/2015	08/08/2016	*Melaleuca cajuputi* Powell	Tràm lá dài	Hà Nội	Msc. Nghiem Duc Trong	01	HNIP/18284/16
36	TD_43	Le Thanh Binh	07/2015	08/08/2016	*Alpinia galanga* (L.) Willd.	Riềng	Hà Nội	Msc. Nghiem Duc Trong	01	HNIP/18285/16
37	TD_44	Le Thanh Binh	08/2015	08/08/2016	*Curcuma zedoaria* (Christm.) Roscoe	Nghệ đen	Hà Nội	Msc. Nghiem Duc Trong	01	HNIP/18286/16

Hanoi, 8th September, 2016

Sender

Le Thanh Binh

Herbarium Keeper

Nghiem Duc Trong
