# Supplementary material for: In Vitro Anti-Leishmanial Activity of Essential Oils Extracted from Vietnamese Plants
Source: Molecules. 2017 Jun 27;22(7):1071. doi: 10.3390/molecules22071071 (PMC6152080; doi:10.3390/molecules22071071)
Supplement: Supplementary file 1 [file molecules-22-01071-s001.zip › Supplementary materials/Identification of scientific name of plants.pdf]

HANOI UNIVERSITY OF PHARMACY, VIETNAM
DEPARTMENT OF BOTANY
****

IDENTIFICATION OF SCIENTIFIC NAME OF PLANTS

Number: 30/2016

No.	Sign	Collector & Sender	Date of collection	Scientific name	Family	Vietnamese name	Locality
1	TD_01	Le Thanh Binh	05/2015	*Melaleuca alternifolia* (Maiden & Betch) Cheel	Myrtaceae	Tràm Úc	Hà Nội
2	TD_02	Le Thanh Binh	05/2015	*Ocimum gratissimum* L.	Lamiaceae	Hương nhu trắng	Hà Nội
3	TD_03	Le Thanh Binh	05/2015	*Pluchea indica* (L.) Less.	Asteraceae	Cúc tần	Hà Nội
4	TD_04	Le Thanh Binh	05/2015	*Platycladus orientalis* (L.) Franco	Cupressaceae	Trắc bách	Hà Nội
5	TD_05	Le Thanh Binh	06/2015	*Piper sarmentosum* Roxb.	Piperaceae	Lá lốt	Hà Nội
6	TD_06	Le Thanh Binh	08/2015	*Artemisia annua* L.	Asteraceae	Thanh hao hoa vàng	Hà Nội
7	TD_07	Le Thanh Binh	05/2015	*Ocimum tenuiflorum* L.	Lamiaceae	Hương nhu tía	Hà Nội
8	TD_08	Le Thanh Binh	08/2015	*Dysphania ambrosioides* (L.) Mosyakin & Clemant	Chenopodiaceae	Dầu giun	Thanh Hoá
9	TD_09	Le Thanh Binh	05/2015	*Blumea lanceolaria* (Roxb.) Druce	Asteraceae	Xương sông	Thanh Hoá
10	TD_10	Le Thanh Binh	05/2015	*Eucalyptus camaldulensis* Dehnh.	Myrtaceae	Bạch đàn trắng	Hà Nội
11	TD_11	Le Thanh Binh	2015	*Litsea cubeba* (Lour.) Pers.	Lauraceae	Màng tang	Quảng Nam
12	TD_12	Le Thanh Binh	06/2015	*Kaempferia galanga* L.	Zingiberaceae	Địa liền	Hà Nội
13	TD_13	Le Thanh Binh	07/2015	*Zingiber zerumbet* (L.) Roscoe ex Sm.	Zingiberaceae	Gừng gió	Hà Nội
14	TD_14	Le Thanh Binh	08/2015	*Ageratum conyzoides* (L.) L.	Asteraceae	Cây cứt lợn	Thanh Hoá
15	TD_18	Le Thanh Binh	07/2015	*Curcuma longa* L.	Zingiberaceae	Nghệ	Hà Nội

No.	Sign	Collector & Sender	Date of collection	Scientific name	Family	Vietnamese name	Locality
16	TD_19	Le Thanh Binh	07/2015	*Pogostemon cablin* (Blanco) Benth.	Lamiaceae	Hoắc hương	Hưng Yên
17	TD_20	Le Thanh Binh	07/2015	*Hyptis suaveolens* (L.) Poit.	Lamiaceae	É lớn tròng	Vĩnh Phúc
18	TD_22	Le Thanh Binh	05/2015	*Vitex trifolia* L.	Verbenaceae	Mạn kinh	Hà Nội
19	TD_23	Le Thanh Binh	08/2015	*Clausena indica* (Dalzell) Oliv.	Rutaceae	Mác mật	Thái Nguyên
20	TD_24	Le Thanh Binh	08/2015	*Elsholtzia blanda* (Benth.) Benth.	Lamiaceae	Kinh giới núi	Lào Cai
21	TD_25	Le Thanh Binh	08/2015	*Elsholtzia penduliflora* W. W. Sm.	Lamiaceae	Chùa dù	Lào Cai
22	TD_26	Le Thanh Binh	08/2015	*Litsea cubeba* (Lour.) Pers.	Lauraceae	Màng tang	Lào Cai
23	TD_27	Le Thanh Binh	2015	*Illicium verum* Hook. f.	Illiciaceae	Hồi	Lạng Sơn
24	TD_28	Le Thanh Binh	07/2015	*Zingiber officinale* Roscoe	Zingiberaceae	Gừng	Hà Nội
25	TD_29	Le Thanh Binh	08/2015	*Hedychium coronarium* J.Koenig	Zingiberaceae	Ngải tiên trắng, Bạch yến	Hà Nội
26	TD_30	Le Thanh Binh	07/2015	*Coriandrum sativum* L.	Apiaceae	Mùi	Hà Nội
27	TD_32	Le Thanh Binh	07/2015	*Anethum graveolens* L.	Apiaceae	Thì là	Hà Nội
28	TD_33	Le Thanh Binh	07/2015	*Plectranthus amboinicus* (Lour.) Spreng.	Lamiaceae	Húng chanh	Phú Thọ
29	TD_34	Le Thanh Binh	08/2015	*Elsholtzia ciliata* (Thunb.) Hyl. *(Syn. = Elsholtzia cristata* Willd.)	Lamiaceae	Kinh giới	Hà Nội
30	TD_35	Le Thanh Binh	08/2015	*Zingiber montanum* (J.Koenig) Link ex A.Dietr.	Zingiberaceae	Gừng núi	Hà Nội
31	TD_36	Le Thanh Binh	08/2015	*Amomum schmidtii* (K.Schum.) Gagnep.	Zingiberaceae	Sa nhân hồi	Quảng Ninh
32	TD_38	Le Thanh Binh	11/2014	*Elsholtzia communis* (Collett & Hemsl.) Diels	Lamiaceae	Kinh giới Đồng Văn	Hà Giang
33	TD_39	Le Thanh Binh	08/2015	*Amomum aromaticum* Roxb.	Zingiberaceae	Thảo quả	Lào Cai

No.	Sign	Collector & Sender	Date of collection	Scientific name	Family	Vietnamese name	Locality
34	TD_40	Le Thanh Binh	08/2015	*Cinnamomum cassia* (L.) J.Presl	Lauraceae	Quế	Quảng Ninh
35	TD_42	Le Thanh Binh	08/2015	*Melaleuca cajuputi* Powell	Myrtaceae	Tràm lá dài	Hà Nội
36	TD_43	Le Thanh Binh	07/2015	*Alpinia galanga* (L.) Willd.	Zingiberaceae	Riềng	Hà Nội
37	TD_44	Le Thanh Binh	08/2015	*Curcuma zedoaria* (Christm.) Roscoe	Zingiberaceae	Nghệ đen	Hà Nội

-

Hanoi, 8th September, 2016

Head of Department

Professor Tran Van On

Determiner

Msc. Nghiem Duc Trong

References

1. Delin Wu, Kai Larsen (2000), Zingiberaceae, in: Wu, Z. Y. & P. H. Raven (eds.*), Flora of China, Vol. 24 (Flagellariaceae through Marantaceae)*, Science Press, Beijing, and Missouri Botanical Garden Press, St. Louis.

2. Dianxiang Zhang, Thomas G. Hartley, David J. Mabberley (2008), Rutaceae, in: Wu, Z. Y., P. H. Raven & D. Y. Hong (eds.), *Flora of China. Vol. 11 (Oxalidaceae through Aceraceae)*, Science Press, Beijing, and Missouri Botanical Garden Press, St. Louis.

3. Gelin Zhu et al. (2003), Chenopodiaceae, in: Wu, Z. Y., P. H. Raven & D. Y. Hong (eds.), *Flora of China, Vol. 5 (Ulmaceae through Basellaceae)*, Science Press, Beijing, and Missouri Botanical Garden Press, St. Louis.

4. Jie Chen, Lyn A. Craven (2007), Myrtaceae, in: Wu, Z. Y., P. H. Raven & D. Y. Hong (eds.), *Flora of China, Vol. 13 (Clusiaceae through Araliaceae)*, Science Press, Beijing, and Missouri Botanical Garden Press, St. Louis.

5. Le Kim Bien (2007) Asteraceae in: Nguyen Tien Ban et al (eds.), *Flora of Vietnam, Volume 7*, Science and Technics Publishing House, Hanoi

6. Liguo Fu et al. (1999), Cupressaceae, in: Wu, Z. Y. & P. H. Raven (eds.), *Flora of China, Vol. 4 (Cycadaceae through*

Fagaceae), Science Press, Beijing, and Missouri Botanical Garden Press, St. Louis.

7. Menglan She et al. (2005), Apiaceae, in: Wu, Z. Y., P. H. Raven & D. Y. Hong (eds.), *Flora of China, Vol. 14 (Apiaceae through Ericaceae)*, Science Press, Beijing, and Missouri Botanical Garden Press, St. Louis.

8. Nianhe Xia, Richard M. K. Saunders (2008), Illiciaceae, in: Wu, Z. Y., P. H. Raven & D. Y. Hong (eds.), *Flora of China, Vol. 7 (Menispermaceae through Capparaceae)*, Science Press, Beijing, and Missouri Botanical Garden Press, St. Louis.

9. Pham Hoang Ho (1999-2000), *Vietnam Plants (An Illustrated Flora of Vietnam), Volume 1-3*, Youth Publishing House, Ho Chi Minh City

10. Shou-liang Chen, Michael G. Gilbert (1994), Verbenaceae, in: Wu, Z. Y. & P. H. Raven (eds.), *Flora of China, Vol. 17 (Verbenaceae through Solanaceae)*, Science Press, Beijing, and Missouri Botanical Garden Press, St. Louis.

11. Shugang Li et al. (2008), Lauraceae, in: Wu, Z. Y., P. H. Raven & D. Y. Hong, (eds.), *Flora of China, Vol. 7 (Menispermaceae through Capparaceae)*, Science Press, Beijing, and Missouri Botanical Garden Press, St. Louis.

12. Vo Van Chi (2004), *Dictionary of common plants, Volume 1-2*, Science and Technics Publishing House, Hanoi

13. Vu Xuan Phuong (2000), Lamiaceae, in: Nguyen Tien Ban et al (eds.), *Flora of Vietnam, Volume 2*, Science and Technics Publishing House, Hanoi

14. Vu Xuan Phuong (2007), Verbenaceae, in: Nguyen Tien Ban et al (eds.), *Flora of Vietnam, Volume 6*, Science and Technics Publishing House, Hanoi

15. Xi-wen Li, Ian C. Hedge (1994), Lamiaceae, in: Wu, Z. Y. & P. H. Raven (eds.), *Flora of China, Vol. 17 (Verbenaceae through Solanaceae)*, Science Press, Beijing, and Missouri Botanical Garden Press, St. Louis.

16. Yung-chien Tseng, Nianhe Xia, Michael G. Gilbert (1999), Piperaceae, in: Wu, Z. Y. & P. H. Raven (eds.), *Flora of China, Vol. 4 (Cycadaceae through Fagaceae)*, Science Press, Beijing, and Missouri Botanical Garden Press, St. Louis.

17. Zhu Shi et al. (2011), Asteraceae (Compositae), in: Wu, Z. Y., Raven, P. H. & Hong, D. Y. (eds.), *Flora of China, Volume 20-21 (Asteraceae)*, Science Press, Beijing, and Missouri Botanical Garden Press, St. Louis.
